# IClinfMRI Software for Integrating Functional MRI Techniques in Presurgical Mapping and Clinical Studies

**DOI:** 10.3389/fninf.2018.00011

**Published:** 2018-03-09

**Authors:** Ai-Ling Hsu, Ping Hou, Jason M. Johnson, Changwei W. Wu, Kyle R. Noll, Sujit S. Prabhu, Sherise D. Ferguson, Vinodh A. Kumar, Donald F. Schomer, John D. Hazle, Jyh-Horng Chen, Ho-Ling Liu

**Affiliations:** ^1^Department of Imaging Physics, The University of Texas MD Anderson Cancer Center, Houston, TX, United States; ^2^Graduate Institute of Biomedical Electronics and Bioinformatics, National Taiwan University, Taipei, Taiwan; ^3^Department of Diagnostic Radiology, The University of Texas MD Anderson Cancer Center, Houston, TX, United States; ^4^Graduate Institute of Humanities in Medicine, Taipei Medical University, Taipei, Taiwan; ^5^Section of Neuropsychology, Department of Neuro-Oncology, The University of Texas MD Anderson Cancer Center, Houston, TX, United States; ^6^Department of Neurosurgery, The University of Texas MD Anderson Cancer Center, Houston, TX, United States

**Keywords:** functional magnetic resonance imaging (fMRI), presurgical mapping, preoperative mapping, resting state, cerebrovascular reactivity, software, interactive, visualization

## Abstract

Task-evoked and resting-state (rs) functional magnetic resonance imaging (fMRI) techniques have been applied to the clinical management of neurological diseases, exemplified by presurgical localization of eloquent cortex, to assist neurosurgeons in maximizing resection while preserving brain functions. In addition, recent studies have recommended incorporating cerebrovascular reactivity (CVR) imaging into clinical fMRI to evaluate the risk of lesion-induced neurovascular uncoupling (NVU). Although each of these imaging techniques possesses its own advantage for presurgical mapping, a specialized clinical software that integrates the three complementary techniques and promptly outputs the analyzed results to radiology and surgical navigation systems in a clinical format is still lacking. We developed the Integrated fMRI for Clinical Research (IClinfMRI) software to facilitate these needs. Beyond the independent processing of task-fMRI, rs-fMRI, and CVR mapping, IClinfMRI encompasses three unique functions: (1) supporting the interactive rs-fMRI mapping while visualizing task-fMRI results (or results from published meta-analysis) as a guidance map, (2) indicating/visualizing the NVU potential on analyzed fMRI maps, and (3) exporting these advanced mapping results in a Digital Imaging and Communications in Medicine (DICOM) format that are ready to export to a picture archiving and communication system (PACS) and a surgical navigation system. In summary, IClinfMRI has the merits of efficiently translating and integrating state-of-the-art imaging techniques for presurgical functional mapping and clinical fMRI studies.

## Introduction

Functional magnetic resonance imaging (fMRI) based on a blood oxygenation level–dependent (BOLD) mechanism has growing significance in clinical imaging studies for the management of neurological diseases (Matthews et al., 2006). One of the promising applications of clinical fMRI is to aid presurgical planning in order to maximize the lesion resection while preventing post-operative functional deficits (Vlieger et al., 2004; Sanai et al., 2008). With use of block-design paradigms, fMRI activations have been shown to correlate well with the clinical gold-standard—intra-operative direct cortical stimulation (DCS)—making it valuable for presurgical planning (Bizzi et al., 2008; Weng et al., 2017). However, functional brain mapping that exploits the task-fMRI technique alone presents two major challenges in patients with intracranial pathology. First, the impaired cerebrovascular reactivity (CVR) can decrease sensitivity and specificity on localizing eloquent regions near or within the lesion (Ulmer et al., 2003, 2004; Pillai and Zacà, 2011, 2012) because BOLD-fMRI relies on intact coupling between neural firing and cerebrovascular response (Roy and Sherrington, 1890; Harrison et al., 2002). Thus, CVR mapping has been recommended as an essential component when the reliability of fMRI mapping is a concern (Pillai and Mikulis, 2015; Pak et al., 2017). Second, since the effectiveness of task-fMRI depends highly on patient's performance and participation, ensuring adequate task compliance can be challenging in patients with neurological deficits or altered behavior capabilities (Pujol et al., 1998; Bookheimer, 2007). As an alternative to task-fMRI, resting-state fMRI (rs-fMRI) has become a promising technique in localizing brain regions in functional networks, regardless of task engagement (Quigley et al., 2001; Zhang et al., 2009; Mitchell et al., 2013). To effectively translate the aforementioned techniques for presurgical planning and fMRI studies in clinical populations, specialized clinical software is required to integrate these complementary techniques and promptly generate useful information in a clinical format before surgery or before a clinical decision is made.

Since the BOLD signal is based on neurovascular coupling, the abnormal cerebral vasculature or regional hemodynamic disruption caused by intracranial pathology can impair the CVR and invalidate the assumption of neurovascular coupling. Such neurovascular uncoupling (NVU) can potentially result in false negative errors in fMRI mapping (lack of BOLD signal despite neural activity) which may potentially contribute to an undesirable resection of eloquent cortex (Ulmer et al., 2003; Pillai and Mikulis, 2015; Pak et al., 2017). Without further confirmation by intraoperative DCS, this fMRI false negative could lead to permanent postoperative neurological deficits. Impaired CVR has been reported in brain tumors (Hsu et al., 2004; Pillai and Zacà, 2012), cerebrovascular diseases (Mikulis et al., 2005; Chang et al., 2013), and neurodegenerative diseases (Iadecola, 2004). In these cases, mapping CVR with MRI during a vasodilatation challenge would be a useful technique to indicate the NVU potential for assisting the interpretation of clinical fMRI activation (Pillai and Mikulis, 2015; Pak et al., 2017), to detect a vascular risk in Alzheimer's disease (Glodzik et al., 2013), as well as to predict early perfusion change after vascular intervention (Chang et al., 2009). Practically, CVR experiments can be conducted by using ordinary fMRI acquisition methods during a breath-holding (BH) task (Kastrup et al., 2001; Liu et al., 2002). The post-processing resembles task-fMRI with several modifications such as the selection of impulse response functions (Birn et al., 2008; Pillai and Zacà, 2012; Jahanian et al., 2016) and the consideration of hemodynamic delays (Birn et al., 2008; Jahanian et al., 2016). Therefore, despite its importance in clinical applications, implementation of MRI data analysis for CVR mapping requires modifications to the existing fMRI software and is a time-consuming procedure that can be difficult without the assistance of an expert.

The rs-fMRI is capable of mapping intrinsic functional networks in which the within-network spontaneous BOLD oscillations emerge in synchrony during rest (Biswal et al., 1995). The rs-fMRI has been shown to be of importance in neurosurgical applications (Lang et al., 2014) and in charactering the integrity of the brain network for a wide variety of diseases (Lee et al., 2013; Matthews and Hampshire, 2016). These networks are often detected with use of seed-correlation analysis (SCA) (Biswal et al., 1995; Shimony et al., 2009) or data-driven approaches such as independent component analysis (ICA) (Smith et al., 2009; Zhang et al., 2009). The SCA approach is straightforward and imposes prior knowledge for seed selection; however, spatial distortions and functional reorganization due to brain lesions can make seed selection difficult on the basis of anatomical landmarks alone. In contrast, although the ICA approach does not have the issues associated with seed placement, determining the proper number of components and selecting components of interest in this approach is challenging (Branco et al., 2016). Software toolboxes have been developed for analyzing rs-fMRI data for presurgical fMRI mapping using either SCA or ICA approaches (Böttger et al., 2011; Huang et al., 2016). However, to the best of our knowledge, no existing software provides integrated visualization, such as an interface that allows the use of task-fMRI results to guide the SCA in rs-fMRI processing and directly generates results that are ready for exporting to a radiology picture archiving and communication system (PACS) and to a neurosurgical navigation system.

Although the aforementioned analyses for various fMRI modalities are feasible with existing research software such as AFNI (Cox, 1996), SPM (Welcome Department of Cognitive Neurology, Institute of Neurology, London, UK), and FSL (Smith et al., 2004) or in-house scripts, a clinical translation of these techniques in a holistic way is needed. In our study, we developed Integrated fMRI for Clinical Research (IClinfMRI) software to facilitate clinical fMRI research with applicability in presurgical fMRI planning. Beyond the independent processing of task-fMRI, rs-fMRI, and CVR mapping, IClinfMRI supports interactive rs-fMRI mapping while visualizing task-fMRI results as a guidance map, provides visualization of sites with potential NVU in fMRI results, and exports overlays of mapping results on structural MR images in presentations that can be readily sent to a clinical PACS as well as to a surgical navigation system.

## Materials and methods

### Developing environment

The IClinfMRI software was developed on the MATLAB 2014a platform (The MathWorks, Inc., Natick, MA, USA). The software was built upon in-house scripts and calls functions in free for noncommercial-use software such as dcm2nii (https://www.nitrc.org/projects/dcm2nii/), AFNI (version 16.2.09) (Cox, 1996), and SPM12 (v6685) (Welcome Department of Cognitive Neurology, Institute of Neurology, London, UK). The dcm2nii is used to convert images from Digital Imaging and Communications in Medicine (DICOM) to Neuroimaging Informatics Technology Initiative (NIfTI) formats. Functions in both AFNI and SPM12 are adopted for data analysis.

### Workflow and processing pipeline

**Figure 1:**
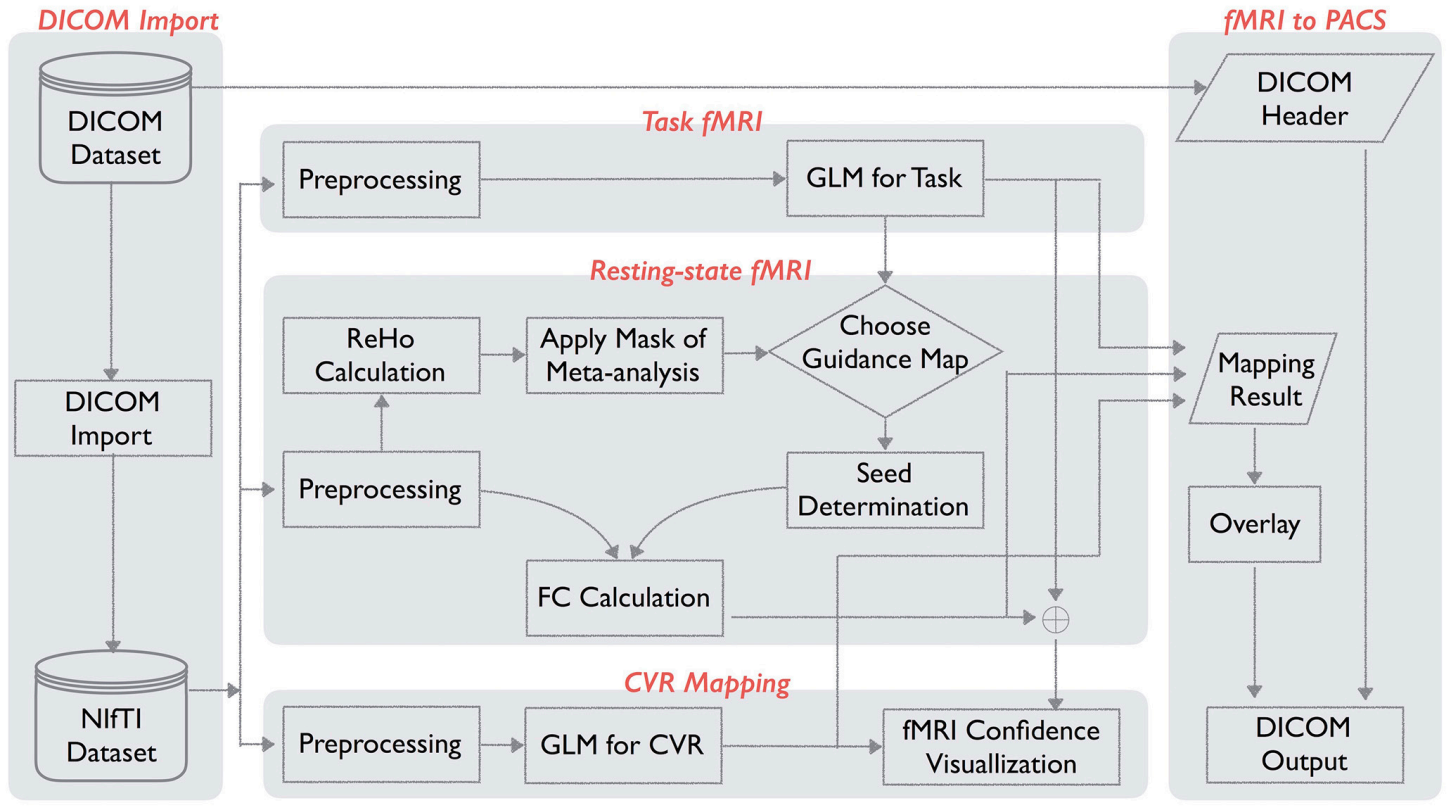
Workflow for integrated fMRI for Clinical Research (IClinfMRI) software.

The IClinfMRI software has five modules: *DICOM Import, Task fMRI, Resting-state fMRI, CVR mapping*, and *fMRI to PACS*. Figure 1 shows the schematic workflow of IClinfMRI and the functionalities provided in each module. In *DICOM Import*, DICOM images in a file folder are recognized, sorted, and converted to NIfTI files, which facilitates interoperability among research image processing software, and is saved in organized subdirectories that are named according to the series descriptions stored in the DICOM header. Next, task-fMRI, rs-fMRI, and BH-MRI data are analyzed by modules of *Task fMRI, Resting-state fMRI*, and *CVR mapping*, respectively. After the analysis, the mapping results in NIfTI format are properly thresholded and overlaid on clinical structural MR images (e.g., T_1_-weighted [T_1_w] or fluid-attenuated inversion recovery [FLAIR] images). The color-coded and gray-scale overlays are exported as a series of DICOM files by using the *fMRI to PACS* module. Table 1 lists the available functions provided in each module. All temporal analyses and image registration call AFNI's functions, and image segmentation and inverse normalization (from template to individual space) call functions from SPM12. Key arguments specified in the functions are described below.

**Table 1:** Summary of IClinfMRI models and functions used to process the fMRI data.

	**Function**	**Module**					**Software (Function)**
		**DICOM Import**	**Task fMRI**	**Resting-state fMRI**	**CVR Mapping**	**fMRI to PACS**	
Data input	DICOM to NIfTI	✓					dcm2nii
Preprocessing	Slice timing		✓	✓	✓		AFNI (3dTshift)
	Motion correction		✓	✓	✓		AFNI (3dvolreg)
	Aligning EPI to T_1_ by coordinate-based registration		✓	✓	✓		AFNI (3dresample)
	Aligning EPI to T_1_ by boundary-based registration		✓	✓	✓		AFNI(align_epi_anat.py)
	Aligning EPI to T_1_ by intensity-based registration		✓	✓	✓		AFNI (align_epi_anat.py)
	De-spiking & Detrending		✓	✓	✓		AFNI (3dDespike; 3dDetrend)
	Nuisance Regression			✓			AFNI (3dBandpass)
	Segmentation		✓	✓	✓		SPM12 (Segmentation)
	Bandpass filtering			✓			AFNI (3dBandpass)
	Smoothing		✓	✓	✓		AFNI (3dmerge)
Detection	HRF-based GLM		✓				AFNI (3dDeconvolve & 3dREMLfit)
	Seed selection on the graph			✓			In-house script
	Seed preview on the graph			✓			In-house script
	Pearson correlation			✓			AFNI (3dfim+)
	Seed-based GLM			✓			AFNI (3dDeconvolve & 3dREMLfit)
	RRF-based GLM with varying latency				✓		AFNI (3dDeconvolve)
	HRF-based GLM with varying latency				✓		AFNI (3dDeconvolve) & SPM12 (HRF)
Display	Adjustable threshold, window, and color map			✓	✓	✓	In-house script
	Fusion of both fMRI and CVR maps on anatomical images				✓		In-house script
Data output	NIfTI to DICOM					✓	In-house script

*DICOM, Digital Imaging and Communications in Medicine; GLM, general linear model; NIfTI, Neuroimaging Informatics Technology Initiative; CVR, cerebrovascular reactivity; HRF, hemodynamic response function; RRF, respiratory response function*.

Functional images are motion-corrected to a reference volume with use of rigid-body registration and then resampled to an isotropic grid matching the orientation of a resampled T_1_w image, which is resampled to the same isotropic grid in advance. A reference volume is the time point from fMRI data that has the fewest outliers, which are defined as the time point with its value deviant from the trend above a limit and are calculated with use of 3dToutcount. In a typical range of 50–500 time points per session, this deviation limit calculated by the default setting is approximately 5.5 × MAD away from the fitted trend, where MAD is median absolute value of time series minus trend. For aligning functional images into a structural T_1_w image volume, three methods are incorporated: coordinate-based registration (CBR), intensity-based registration (IBR), and boundary-based registration (BBR). For the CBR method, the spatial resolution of fMRI dataset is resampled to match that of a T_1_w image using 3dresample. For the IBR method, the alignment matrix is estimated by a reference volume of the functional data to a T_1_w image by using the cost function of normalized mutual information (align_epi_anat.py with the option of “-dset2to1” and “-cost nmi”). For the BBR method, a high-resolution echo-planar imaging (EPI) volume with prominent tissue boundaries of gray matter (GM), white matter (WM), and cerebrospinal fluid (CSF) is required and registered to a T_1_w image using the cost function of local Pearson correlation (align_epi_anat.py with the option “-epi2anat”) (Saad et al., 2009). In the study that proposed the BBR method (Saad et al., 2009), the spatial resolutions were 3 × 3 × 3 mm^3^ and 1 × 1 × 1 mm^3^ for echo-planar and T_1_w images, respectively, which led to an improved registration as compared to CBR and IBR. When the users prefer BBR but their fMRI datasets are acquired at a lower resolution and the resultant echo-planar images do not have clear tissue boundaries, our software provides the option for them to use an additionally acquired single-volume high-resolution echo-planar image to determine the spatial transformation. The reference volume of the functional image data is then co-registered to the high-resolution EPI image (align_epi_anat.py with the option of “-dset2to1” and “-cost nmi”), and a concatenated transformation matrix is obtained from the two-step process. For the IBR and BBR alignment, their transformation matrix is combined with the one for motion correction; therefore, spatial transformation and image interpolation (using *wsinc5*, 3dAllineate) are performed only once for each original functional image volume. The final voxel size of aligned functional images is 2-mm or 3-mm isotropic, as determined by users.

Task-fMRI activation maps are generated with use of the general linear model (GLM) by calculating the fitness of the preprocessed fMRI signal to the expected response that was constructed by convolving a canonical hemodynamic response function (HRF) with the task paradigm. During the fitting procedure, six motion parameters generated in the preprocessing step are set as the nuisance regressors, and the temporal autocorrelation structure in the residual is corrected by using the generalized least squares technique (3dREMLfit) with an autoregressive model of order 1 and moving average model of order 1, i.e., ARMA(1,1).

For rs-fMRI analysis, noise components are modeled by six motion parameters and two averaged signal fluctuations over masks of WM and CSF. These masks are generated by setting a probability threshold of 99.9% on the segments of the three-dimensional (3D) T_1_w image. The 3D T_1_w image was segmented into GM, WM, and CSF tissue segments using unified segmentation approach (Ashburner and Friston, 2005) in SPM12. On the basis of the probabilistic framework, this tool involved circular procedure of image registration, tissue classification, and bias field correction for optimizing the segmentation. Both masks are eroded by one voxel along each of the three axes (Jo et al., 2010) for preventing the partial volume effect on the masks and then resampled to match the spatial resolution of the aligned rs-fMRI data. It is optional to include a global signal fluctuation as an additional noise component by extracting the averaged signal from a whole brain mask produced by setting a sum of tissue probability maps of GM, WM, and CSF with a threshold of 30%. After removing the noise components and performing other preprocessing steps, such as the temporal filtering (Table 1), the rs-functional connectivity (FC) map is obtained by correlating the average time series from a seed region against that from every voxel in the brain.

### Unique features

Instead of analyzing each fMRI modality independently, IClinfMRI provides a unique workflow to integrate them for clinical research applications. In the *Resting-state fMRI* module, we designed a double-panel graphical user interface (GUI) to support interactive rs-fMRI mapping while visualizing a guidance map of the user's choice for seed placement. The guidance map can be an anatomical image, a functional map processed by IClinfMRI's *Task fMRI* module, or a parametric map resulting from other research software. In this module, the seed is placed by using a simple mouse click on either of the two panels, and the seed-based FC map will be calculated, converted to a Fisher's z map, and then interactively updated on the bottom right panel of the GUI. In addition, visualization of mapping results can be easily manipulated with options that include window, threshold, opacity, and color map. Two approaches are suggested and implemented to obtain a guidance map: (1) processing task-fMRI data by using the *Task fMRI* module, and (2) calculating a regional homogeneity (ReHo) map with the use of 3dReHo (Taylor and Saad, 2013) embedded in the *Resting-state fMRI* module. The ReHo analysis summarizes the local FC by measuring the temporal similarity between a given voxel and its neighborhood with the use of Kendall coefficient of concordance (Zang et al., 2004). The ReHo map has been proposed as the alternative to guide seed selection and improve the sensitivity of rs-FC network detection (Yan et al., 2013). Besides, local activity could also contain useful information in tumor studies (Duan et al., 2016), but we have not yet incorporated this feature in our software. In our *Resting-state fMRI* module, the preprocessed rs-fMRI data before spatial smoothing was fed into the ReHo analysis for obtaining ReHo map (Zuo et al., 2013). In addition to offering the ReHo map alone, we further implemented a novel approach that seeds the connectivity analysis based on the ReHo map confined within a mask obtained from meta-analysis (RH + MA). For the analysis of language network, the meta-analysis result was downloaded from the Neurosynth (http://neurosynth.org/) by using the term “language” that resulted from 885 studies (Yarkoni et al., 2011) and then inversely normalized the meta-analysis maps from standard space to the native space using SPM12. Since it was corrected for a false discovery rate (FDR) of 0.01, we did not apply an additional threshold but constrained the result within the brain regions covering the putative Broca's and Wernicke's areas. These brain regions included middle frontal gyrus and inferior frontal gyrus for the traditional Broca's area, and comprised angular gyrus, supramarginal gyrus, and superior temporal gyrus for the traditional Wernicke's area, implemented by using the LONI Probabilistic Brain Atlas (Shattuck et al., 2008). The final mask was then dilated for 4-mm to consider the altered functional anatomy in patients.

In the *CVR mapping* module, two types of impulse response functions are implemented, the canonical HRF (Birn et al., 2008; Jahanian et al., 2016) and the respiratory response function (RRF) (Birn et al., 2008; Pillai and Zacà, 2012), to generate the CVR map by using the GLM with an adjustable series of multiple time delays. In the voxel-wise optimization, the GLM analysis is repeated for each time delay, and the maximum *t*-value across multiple GLM results will be selected for each voxel, to account for the varying latency of the CVR responses across the brain (Birn et al., 2008; Jahanian et al., 2016). We provide the visualization for fusions of both fMRI and CVR maps on anatomical images, which integrates the results into a single presentation. This allows the display of areas with potential NVU, that is, potential activated areas with both negative fMRI and CVR, near or within the lesion.

Presenting the mapping result in a clinical format is an essential function in software designed for clinical applications. In IClinfMRI, the *fMRI to PACS* module is used for exporting the functional mapping result to not only color-coded but also gray-scale overlays as DICOM images that can be fed into the same patient directory in PACS and surgical navigation system, respectively. Multiple anatomical images, e.g., T_1_w and FLAIR images, can be selected as underlays, for the same functional overlay to be output in the same series. For color-coded output, three sets of DICOM files are produced, one in each of the three orthogonal planes.

### Software installation and use

IClinfMRI is available as a zip file upon request, and the user can unzip it to a local directory. Under the MATLAB environment, the user can click “Set Path” and add the directory containing IClinfMRI to the path. IClinfMRI will run by simply typing “IClinfMRI” in Matlab. Note that IClinfMRI calls functions in dcm2nii, AFNI, and SPM12; thus, these software packages need to be installed and their functionalities verified before using IClinfMRI.

### Illustrative cases

Analyses of two patients are presented to illustrate the utility of IClinfMRI. Written informed consent was obtained from each patient in accordance with the guidelines and the study protocol approved by the Institutional Review Board at MD Anderson Cancer Center.

#### Patient #1

Patient #1 had a left temporal-occipital lesion with imaging characteristics concerning for a glioblastoma (WHO Grade IV). This dataset was used to demonstrate the rs-fMRI analysis workflow with the seed placement guided by task-fMRI results. Presurgical mapping of speech areas was requested by the neurosurgeon since the tumor was located near the cortical and subcortical language areas, in particular, the posterior part of the language network (the traditional Wernicke's area). Language task-fMRI, rs-fMRI, T_2_w FLAIR, and 3D T_1_w imaging were performed on a 3T clinical scanner (GE Healthcare, Milwaukee, WI, USA). For fMRI acquisition, a ${\text{T}}_{2}^{*}$-weighted gradient-echo EPI (GE-EPI) sequence was used with the following parameters: repetition time (TR)/echo time (TE) = 2000/25 ms, flip angle = 90°, 32 slices with 4-mm thickness and no gap, in-plane resolution = 3.75 mm × 3.75 mm, parallel imaging with acceleration factor of 2. The 3D T_1_w image was acquired using a gradient-echo sequence (TR/TE/inversion time = 7.4/2.1/400 ms; flip angle = 20°; 124 slices with 0.94 × 0.94 × 1.2 mm^3^ voxels). Language task-fMRI included a letter fluency paradigm, a category fluency paradigm, and a sentence completion paradigm. A total of 130 image volumes were obtained from each of the first two paradigms, which started with a 20-s rest period, followed by six cycles of 20-s task block and 20-s rest interval. For the sentence completion paradigm, a total of 120 image volumes were obtained without the last 20-s rest interval. For rs-fMRI, patients were asked to keep their eyes closed, not move their head, not fall asleep, and not think of anything in particular. The acquisition period was 6 min, during which a total of 180 volumes were obtained. The task-fMRI data were processed with use of the *Task fMRI* module with the following default procedures: motion correction, aligning to a T_1_w image with a 2-mm isotropic grid via CBR method, de-spiking, spatial smoothing with a 4-mm FWHM 3D Gaussian kernel, and GLM analysis. The rs-fMRI data were first preprocessed in the *Resting-state fMRI* module with the following default procedures: slice timing, motion correction, aligning to a T_1_w image with a 2-mm isotropic grid via the CBR method, de-spiking, detrending, nuisance regression (mask of WM and CSF), band-pass filtering (0.01–0.08 Hz), and spatial smoothing with a 4-mm FWHM 3D Gaussian kernel. Next, seed placement of the rs-fMRI data was guided by a task-fMRI map resulting from the *Task fMRI* module. For this patient, in particular, the seed was positioned at a local maximum of the task-evoked activations near the anterior part of the language network (traditional Broca's area) because the lesion was in left temporal-occipital lobe near the traditional Wernicke's area.

In addition to fMRI, BH-MRI was also performed on the same patient, and the dataset was used to demonstrate the CVR analysis and visualization. For BH-MRI acquisition, a GE-EPI sequence was used with the same setting of fMRI parameters as described above, except that the TR was set to 3,000 ms. The BH paradigm was 210 s long and comprised an initial 30-s natural breathing period, followed by three cycles of alternations between 15-s BH and 45-s natural breathing. Respiration monitoring device was used to confirm the subject's compliance during the BH CVR MRI. The data were preprocessed by using the *CVR Mapping* module with the following setup: motion correction, aligning to a T_1_w image with a 2-mm isotropic grid spatial resolution via the CBR method, de-spiking, and spatial smoothing with a 4-mm FWHM 3D Gaussian kernel. The onset time and the task duration recorded during data acquisition were set as the task paradigm. The CVR map with a BH task was generated by using a GLM model with multiple respiratory delays ranging from −10 to 15 s between the task and expected RRF.

#### Patient #2

Patient #2 had a left temporal-parietal mass with imaging characteristics consistent with glioblastoma (WHO Grade IV). This dataset was used to demonstrate the rs-fMRI analysis workflow when no task-fMRI results were available. According to neuropsychological assessment, the patient showed significant receptive language deficits and global impairment during testing; thus, he was unable to perform a task-fMRI. Instead, the rs-fMRI acquisition was requested to provide presurgical mapping of the eloquent language areas near the lesion. The image protocol included T_1_w imaging, T_2_w FLAIR imaging, and rs-fMRI. The rs-fMRI data processing was similar to that of Patient #1, except for that the seed was guided by the RH + MA map and placed at a local maximum of ReHo in the meta-analysis mask of the anterior portion of the language network.

### Validation of the rs-fMRI workflow

To validate our software and workflow for rs-FC mapping, the rs-fMRI data for the two patients were also processed with the procedure adopted in a previous study (Hart et al., 2016) using AFNI software, independent from the IClinfMRI. The independent processing procedure and parameters were identical to ours except for the band-pass filtering (0.01–0.1 Hz), spatial smoothing (6-mm FWHM), and the diameter of the seed (10 mm).

## Results

**Figure 2:**
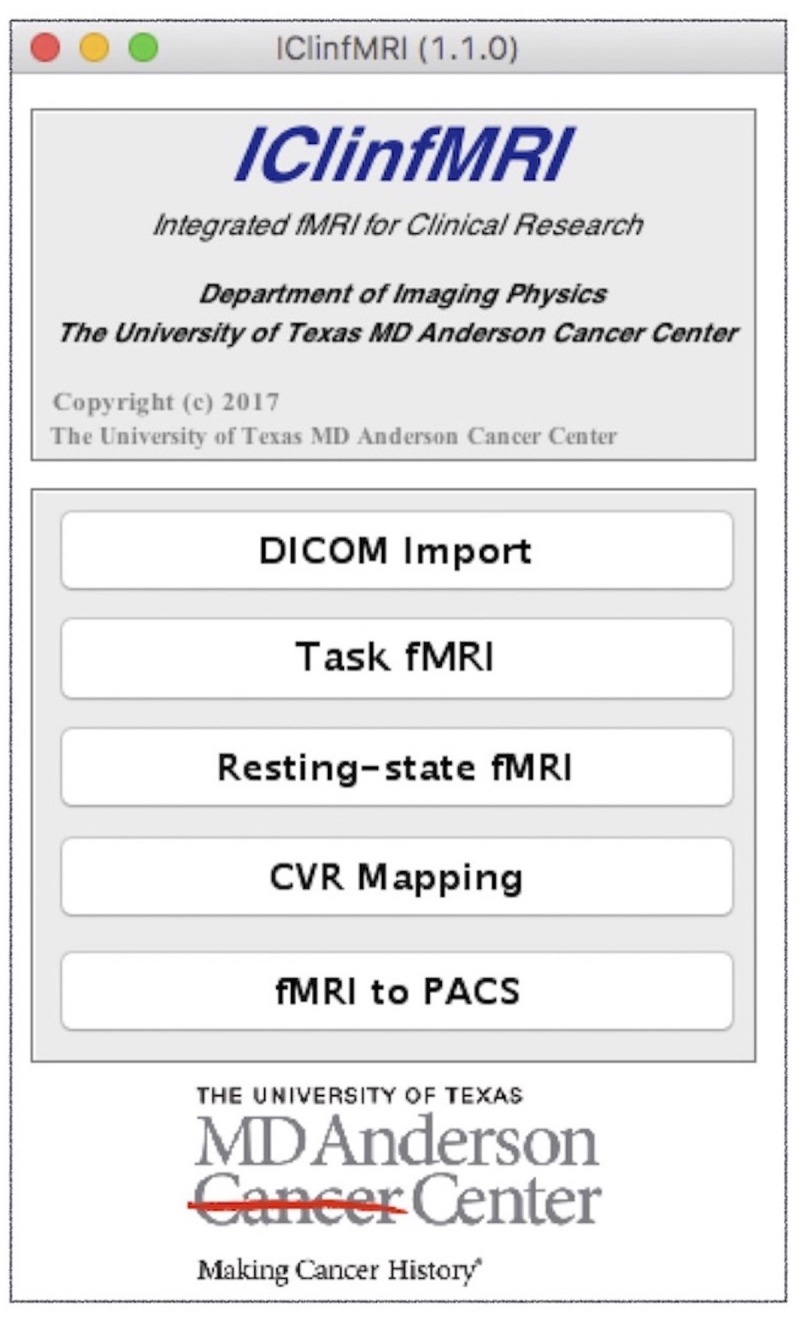
The main graphical user interface (GUI) of IClinfMRI. The main window comprises the five modules of the software.

Typing “IClinfMRI” in the MATLAB command window opens the main GUI window (Figure 2). Users can click on one of the five modules to start corresponding functions of the program.

### DICOM import

As the first module of IClinfMRI, *DICOM Import* requires users to select a main directory, and all subdirectories containing DICOM files will be automatically recognized using MATLAB function “isdicom” and then renamed according to its embedded DICOM information. Concerning the difference in series naming across the center, three options for subdirectories naming are available in “Folder Rename Option” panel of the GUI, including (1) series description, (2) patient's ID and series description, (3) patient's ID, series description, and series number. Next, the DICOM files in various directories are converted to NIfTI format. The converted NIfTI files are used for the following four modules.

### Task fMRI

**Figure 3:**
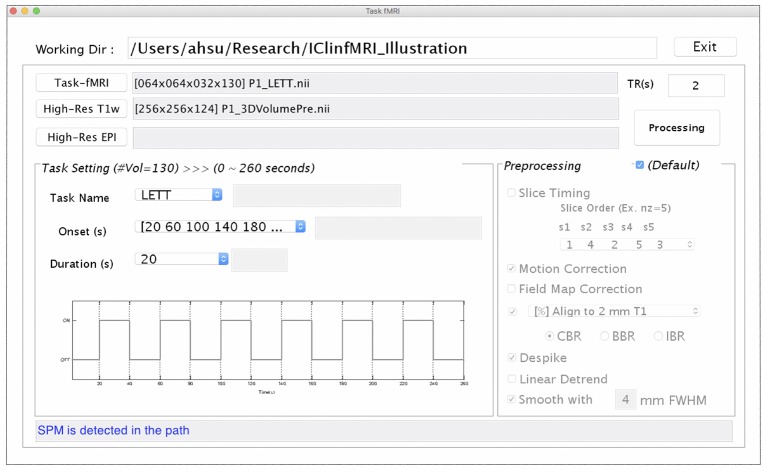
The GUI of task fMRI module. This window consists of the imaging data input **(Upper)**, task paradigm setting **(Bottom Left)**, and preprocessing setting **(Bottom Right)** panels.

Since presurgical planning requires task-fMRI activations to be superimposed on an anatomical image, both task-fMRI and high-resolution T_1_w images are requested in the *Task fMRI* module, using the “Task-fMRI” and “High-Res T1w” button (Figure 3). The “High-Res EPI” is optional. It allows users to select a high-quality echo-planar image volume, when available, to which the BBR algorithm can be applied to improve the registration with the T1w image. After users provide the onset timing and duration of a task in the unit of seconds, the timing of the paradigm will be updated, as shown in Figure 3. Once the required data and task paradigm are both set, the “Processing” button will be enabled, and the “Preprocessing” section can be modified if the checkbox “Default” is unselected. After the “Processing” button is selected, the task-fMRI data will sequentially undergo the selected preprocessing steps, followed by the GLM-based activation detection. The resulting *t*-statistical map is saved in a folder named by the task-fMRI data, which can be output for presurgical planning with the last module as well as retrieved in the *Resting-state fMRI* and *CVR Mapping* modules when needed.

### Resting-state fMRI

**Figure 4:**
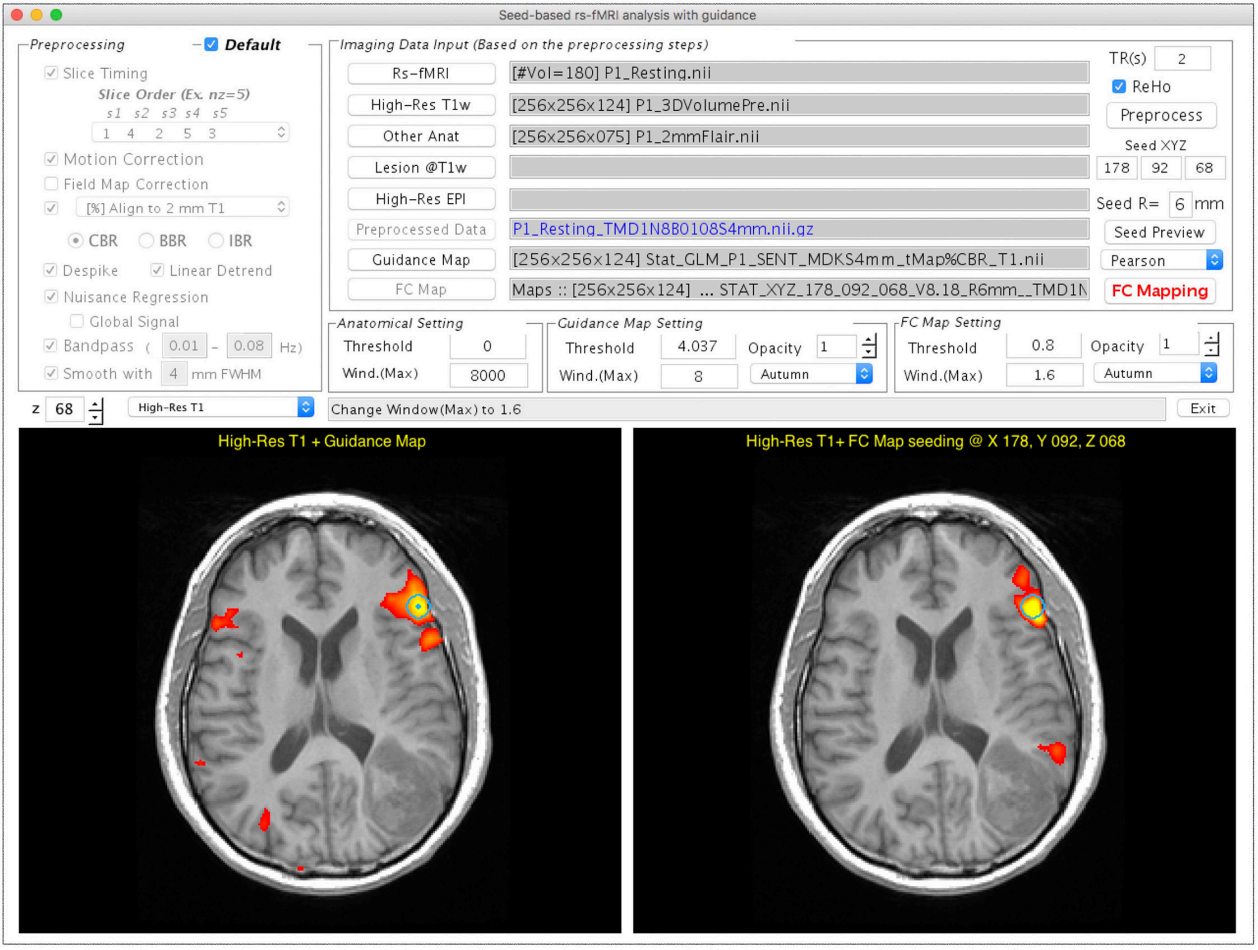
GUI of the *Resting-state fMRI* module displayed with processing and visualization for Patient #1. This window consists of the preprocessing selection **(Upper Left)**, imaging data input panel **(Upper Right)**, visualization widget **(Middle Right)**, and two visualization panels **(Bottom Left** and **Right)**. The **Bottom left** panel shows that the task-fMRI activation overlays (*t* > 4.04, *p* < 10^−4^, uncorrected) on a high-resolution T_1_w image. The **Bottom right** panel shows the FC mapping result obtained with the seed determined based on the task-fMRI (turquoise circle).

Figure 4 demonstrates the user interface of the *Resting-state fMRI* module, with the processing for Patient #1. Both rs-fMRI data and high-resolution T_1_w image are required, whereas the following three are optional inputs in this module. First, the “Other Anat” button was designed to visualize the resulting rs-FC map on another structural image such as a T_2_w FLAIR image. When an image is selected via this button, the image will be aligned to the T_1_w image by using the AFNI “align_epi_anat.py” function with the “-dset2to1” option and “-lpa” cost function. Second, the user can select a binary lesion mask using the “Lesion@T1w” button to exclude the lesion from the WM and CSF masks that are used to generate nuisance regressors in the FC calculation. Third, the utility of the “High-Res EPI” button resembles to *Task fMRI* module. After completing the data selection, one can click “Preprocess” for preprocessing the rs-fMRI data. The ReHo map of the preprocessed rs-fMRI data will be provided if the “ReHo” checkbox is selected. The guidance for seed selection is a unique function that we designed in this module. Users can select the “Guidance Map” button to superimpose the fMRI result, the ReHo results, or the RH + MA results on the displayed structural image for guiding the seed placement. After completing the data preprocessing, users can then define a seed by left-clicking the mouse on the bottom panels of the GUI, adjust the radius of seed sphere by using the edit box following the “R = ” description, view the seed on the GUI by using “Seed Preview” button, and calculate the seed-based FC map by using “FC Mapping” button. In the demonstrated case (Figure 4), a seed (the turquoise dot with circular contour) was positioned on a local maximum (*t* = 8.18, uncorrected *p* < 5 × 10^−13^) of the sentence completion task-fMRI activation near the traditional Broca's area. The resulting rs-FC *z*-map is illustrated in the bottom right panel of the GUI, and the “FC Map Setting” is enabled for adjusting the threshold.

### CVR mapping

**Figure 5:**
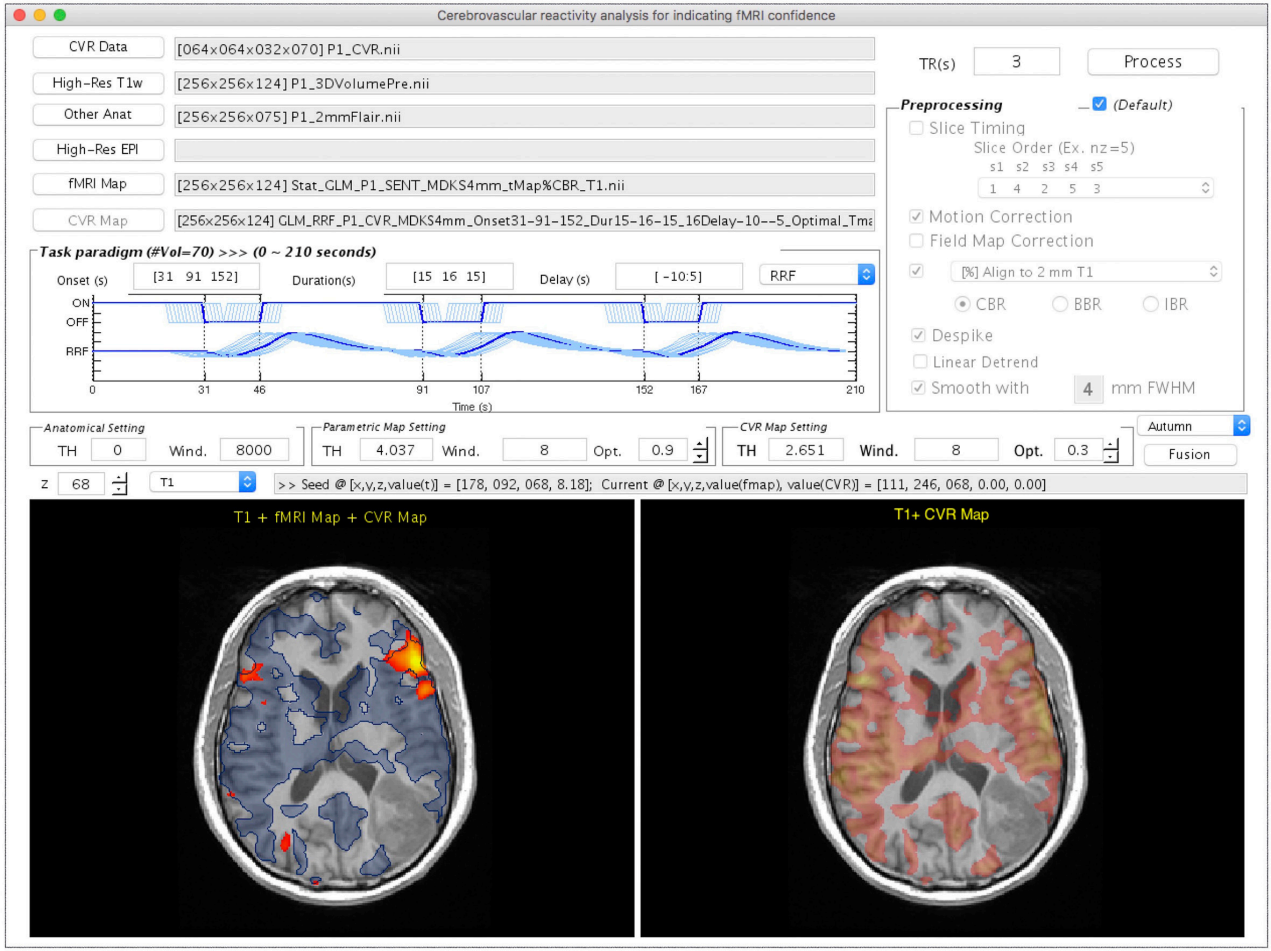
The GUI of the *CVR Mapping* module displayed with processing and visualization for Patient #1. This window consists of the imaging data input panel **(Upper Left)**, breath-hold paradigm setting **(Middle Left)**, preprocessing selection **(Upper Right)**, visualization widget **(Middle)**, and two visualization panels **(Bottom Left** and **Right)**. The **Bottom Left** panel shows the task-fMRI activation overlays (*t* > 4.04, *p* < 10^−4^, uncorrected) on a high-resolution T_1_w image using the warm color map. The result of CVR mapping (*t* > 2.65, *p* < 0.01, uncorrected) displays as transparent blue with a solid blue contour. The area outside the CVR map, but inside potential functional anatomy near the lesion, indicates the location of possible NVU. The **Bottom Right** panel shows the CVR map using a warm color map.

Figure 5 shows the *CVR Mapping* module, illustrated with the processing for Patient #1. Once the CVR data and T_1_w images are selected, adjustments on the following four parameters become available: (1) the onset time of the BH period, (2) the duration of each breath-hold, (3) multiple time delays applied to the GLM analysis, and (4) response function used in the GLM analysis. Note that the flexibilities are given in the onset and duration settings of each BH session, since in practice they usually vary among patients/studies. As the parameters are adjusted, the diagram will be updated in the “Paradigm Setting” panel. Similar to the previous modules, the “Other Anat” and “High-Res EPI” are optional inputs. The “fMRI Map” button is used for selecting the task- or rs-fMRI result to be displayed in the bottom left panel (Figure 5). The option of the preprocessing step for CVR mapping is identical to that in the *Task-fMRI* module (Figure 3). After the processing, the bottom right panel will automatically display the CVR map overlaying on the structural image (Figure 5). Finally, the “Fusion” button is enabled to fuse the resulting CVR map to the preselected fMRI results. Specifically, we display the thresholded CVR map as transparent blue with the solid blue line, together with fMRI activations (or networks) on the anatomical image. In this patient, markedly diminished ipsilateral CVR was seen in the areas within/near the tumor, which indicated NVU potentials and risk of false-negative results in fMRI.

### fMRI to PACS

To convert an fMRI overlay on anatomical images to DICOM files, an anatomical image and parametric map are both required inputs. An option of saving an fMRI overlay on both T_1_w and another anatomical image (e.g., T_2_w FLAIR) in the same series was made available. DICOM headers of images in the resulting series adopted partially those in a DICOM image selected by the user, e.g., one of the original T_1_w DICOM images. The output of this module included a series of DICOM images of white overlay and three series of color-coded overlays in three orthogonal orientations, respectively.

### rs-fMRI case results and validation

**Figure 6:**
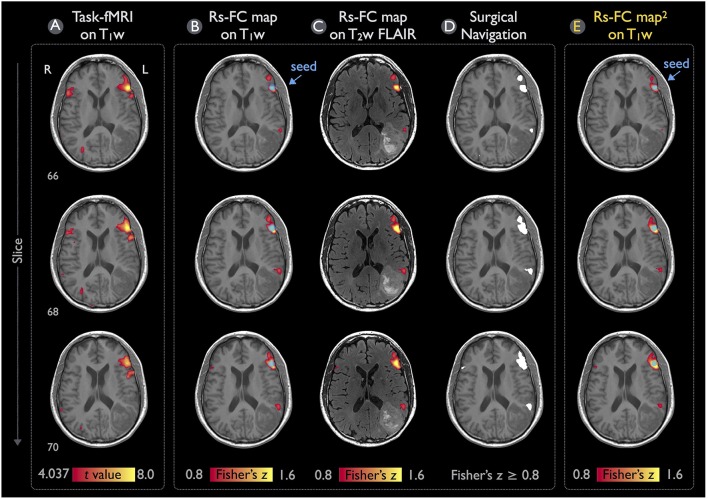
Language mapping resulting from task-fMRI and rs-fMRI for Patient #1. **(A)** The task-fMRI activation above the *t-*statistic threshold of 4.04 (*p* < 10^−4^, uncorrected) was overlaid on the T_1_w image. **(B,C)** Seeding at the peak *t*-value on the task-fMRI activation (blue circle), the rs-FC map above the Fisher's *z* threshold of 0.8 was overlaid on the T_1_w and T_2_w FLAIR images. **(D)** The thresholded rs-FC map overlaid on the T_1_w image in the gray-scale DICOM format. **(E)** The rs-fMRI result analyzed independently from the IClinfMRI by using the procedure adopted in a previous study (Hart et al., 2016).

Figure 6 illustrates the language mappings obtained via task-fMRI and rs-fMRI data for Patient #1. In these speech-fMRI results (Figure 6A), significant activations (*t* > 4.04, *p* < 10^−4^, uncorrected) were detected in bilateral frontal areas and right temporal/parietal area, but not in the left temporal/parietal area or in the traditional Wernicke's area. The rs-fMRI was helpful in that a clear language network between the left frontal and temporal/parietal areas was detected (*z* ≥ 0.8), as demonstrated in Figures 6B,C (the same results but three kinds of overlay/underlay outputs from this module). Note that the gray-scale fused DICOM format (Figure 6D) can be directly imported by surgical navigation software. Moreover, the rs-fMRI result (Figures 6B–D) was consistent with that obtained by using the procedures described in the previous study and software independent from IClinfMRI (Figure 6E).

**Figure 7:**
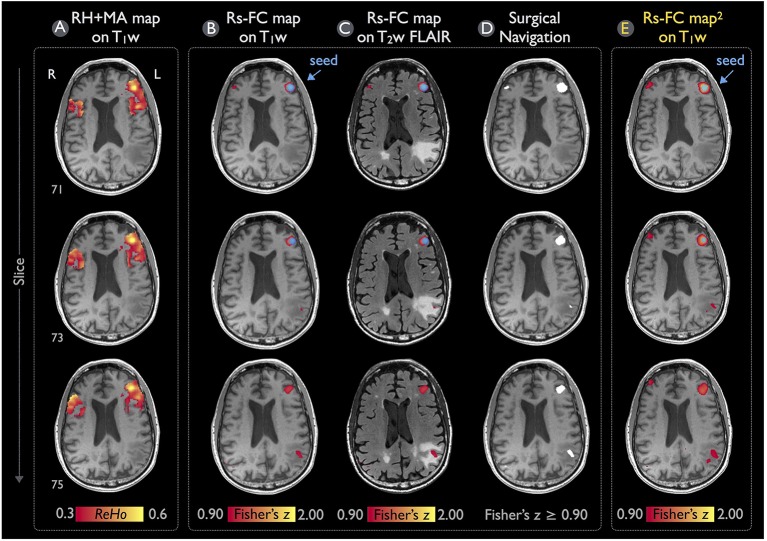
Language mapping of rs-fMRI with seed guided by RH+MA map for Patient #2. **(A)** The RH+MA map with a ReHo threshold of 0.3 is overlaid on the T_1_w image. **(B,C)** Seeding at a local maximum of ReHo value (blue circle), the rs-FC map above the Fisher's *z* threshold of 0.9 is overlaid on the T_1_w and T_2_w FLAIR image. **(D)** The thresholded rs-FC map overlaid on the T_1_w image in the gray-scale DICOM format. **(E)** The rs-fMRI result analyzed independently from the IClinfMRI by using the procedure adopted in the previous study (Hart et al., 2016).

Figure 7 demonstrates the rs-fMRI language mapping results for Patient #2, who was not able to perform the speech fMRI tasks. The RH+MA map showed scattered hot spots in the frontal regions (Figure 7A). The seed placed in the putative Broca's area with a peak ReHo value was able to detect functional connectivity in brain regions close to the tumor, which were suspected to be in the posterior portion of the language network (Figures 7B–D). Similar to the results of Patient #1, the rs-fMRI connectivity pattern found by IClinfMRI was similar to that obtained by using previously published procedures (Figure 7E).

## Discussion

Specialized software, IClinfMRI, was proposed in this study to integrate complementary fMRI techniques in clinical studies with applicability for presurgical planning. Clearly distinguishable from well-established fMRI software package, IClinfMRI was designed with user-friendly modules that can be easily fitted into the clinical workflow. These modules include importing/sorting DICOM images, exporting results that can be recognized by clinical PACS and surgical navigation system, and platforms that can analyze the three major clinical fMRI techniques, namely task-fMRI, rs-fMRI, and CVR mapping, independently yet in an integrated fashion. Unique functionalities of our software include the guidance of seed placement for the interactive rs-fMRI mapping and the visualization of CVR results for indicating potential NVU in fMRI activation maps. The IClinfMRI was specifically designed for translation to clinical fMRI practice with careful validation and is the focus of ongoing research for our team.

For the seed-based FC analysis of rs-fMRI data, previous studies predominantly used anatomical landmarks as guidance in placing a seed (Liu et al., 2009; Shimony et al., 2009; Zhang et al., 2009). The prototype of an interactive toolbox was also developed to calculate and visualize the rs-FC mapping after a seed was placed on an anatomical image (Böttger et al., 2011). However, it is well recognized that the functional localization of brain networks varies in healthy subjects (Mueller et al., 2013), and the variability can be even greater in patients with intracranial pathology (Mitchell et al., 2013). Alternatively, Rosazza et al. placed the seeds on the basis of task-fMRI activation and found that this approach was more sensitive in detecting sensorimotor networks in patients with lesions adjacent to functional areas than were seeds placed on the basis of anatomical landmarks (Rosazza et al., 2014). Cochereau et al. demonstrated 80% accuracy in detecting the rs-language network when seeding at functional sites determined by positive intraoperative DCS results (Cochereau et al., 2016). These studies suggested that determining seeds with guidance from independent functional localizations may improve the results of rs-fMRI analysis. Moreover, Yan et al. proposed a method to use ReHo for assisting seed localization in the rs-fMRI analysis, where the technical logic is described below. When defining a seed with a certain radius (or a volume) for rs-FC analysis, one averages time course across all voxels within this volume to form the reference time curve. Such averaging is based on the assumption that the time curves in those neighboring voxels are well synchronized. However, without examining the resting-state fMRI time curves, one would not know the spatial extent of the well-synchronized regions unless calculating the ReHo index. In other words, the seed selection is still primarily based on anatomical location, like most of the studies using seed-based analysis (Zhang et al., 2009; Rosazza et al., 2014; Hart et al., 2016; Huang et al., 2016), and the ReHo index is only used to refine the group of clusters within/around the assumed anatomical location.

Recently, a software toolkit called *PreSurgMapp* was developed to process both task-fMRI and rs-fMRI, but seed guidance and visualization were not emphasized (Huang et al., 2016). In this work, we designed a double-panel GUI to perform the interactive rs-fMRI mapping while presenting another functional map as guidance for seed determination. Users can directly place a seed in either of the two windows, and it will be synchronously presented in the guidance map and rs-FC results for comparison. This interactive mapping facilitates exploration of the rs-functional network and makes it intuitive to users.

A limitation of our rs-fMRI module is that it supports only seed-based analysis rather than data-driven approaches. Seed-based analysis is more intuitive for users who have clear targets in functional networks that they intent to detect, such as motor and language, which is usually the situation in presurgical mapping. In addition, previous studies indicated the FC maps derived from the seed-based approach have higher consistency with task-fMRI results than do FC maps derived from an independent component analysis (Quigley et al., 2001; Branco et al., 2016; Cochereau et al., 2016; Sair et al., 2016). More recently, methods have been proposed to address the issue of component number optimization for the data-driven analysis (Lu et al., 2017). As the sensitivity and specificity of data-driven approaches improve, we will incorporate these approaches into the IClinfMRI workflow in the future.

A technical challenge of fMRI mapping in patients with neurological diseases is potential false-negative detection due to impaired neurovascular coupling in brain areas adjacent to or within the lesion. This challenge highlights the importance of CVR mapping to indicate brain regions with potential NVU, and subsequently to improve the confidence level of the task-fMRI. However, the existing clinical fMRI software packages, including those designed for presurgical mapping, do not support processing procedures specific to analysis of CVR mapping, such as a varied BH period, respiratory response function, and multiple delays in GLM calculation. Henceforth, we provided a *CVR Mapping* module with these functionalities to fill this gap in clinical needs. In terms of visualization, the double-panel GUI design, as well as the fused display, allows users to synchronously examine both fMRI and CVR mapping results precisely in their relative spatial positions. Thus, the lack of fMRI activation in targeted functional locations but without significant CVR would be a warning of false-negative detection due to the NVU near or within the lesion.

Many research image processing toolboxes and fMRI processing software packages are able to deal with DICOM images. However, exporting the analysis results into clinical PACS and into a surgical navigation system requires not only outputting the results in DICOM format but also writing the DICOM header in a harmonized manner with other images of the same study. For example, original patient and study information should be kept, and a new series number and description should be generated. These aspects are well considered in all software provided by the MRI vendors and in other FDA-cleared software. Although previously developed research toolboxes for presurgical mapping provide the function to analyze fMRI data (Böttger et al., 2011; Huang et al., 2016), the lack of converting data in the DICOM format was the major obstacle for clinical practices. By giving the proper DICOM header, the *fMRI to PACS* module allows users to export a mapping result to the DICOM format by reattaching patient's information. It is important to note that this module can be used to export any images in NIfTI format and is not specifically constrained to the results analyzed by IClinfMRI, which makes it a general tool for wider applications such as quantitative imaging.

Concerning the validity of rs-fMRI functional mapping workflow, we demonstrated that the rs-FC maps processed with the procedure set in IClinfMRI were in accordance with the rs-FC maps processed with the procedure adopted in a previous study (Hart et al., 2016) using AFNI software alone (Figures 6, 7). Individual algorithms adopted in this module were identical to those used in previous validation studies that compared seed-based rs-fMRI with intraoperative mapping (Zhang et al., 2009; Rosazza et al., 2014; Cochereau et al., 2016). Minor differences of these rs-FC maps were expected because their preprocessing procedure and parameters were slightly different (see the Materials and Methods section). For example, a smaller spatial extent in Figure 7B compared with that in Figure 7E was caused by the smaller smooth kernel applied in our procedure because spatial extent has been demonstrated to be directly associated with the spatial smoothing kernel on rs-FC maps (Wu et al., 2011).

In fMRI analysis, spatial smoothing is an important preprocessing step to improve the signal-to-noise ratio (SNR) of the fMRI dataset and a prerequisite for the further statistical analysis using the GLM (Lindquist, 2008). The degrees of spatial smoothing on fMRI dataset have demonstrated a significant impact on task-fMRI mapping (Lu et al., 2012) and rs-fMRI mapping (Wu et al., 2011; Hsu et al., 2016). While the spatial smoothing improves SNR, it sacrifices the spatial resolution of the functional maps. The reduction in spatial resolution may be an undesirable cost for surgical planning. Generally, smoothing kernel size for presurgical fMRI mapping vary between 0 and 8 mm FWHM (Kokkonen et al., 2009; Liu et al., 2009; Lu et al., 2012; Kristo et al., 2014; Huang et al., 2016). Considering the benefit and cost of spatial smoothing, the smoothness of 4-mm FWHM is set as the default based on our experiences. Furthermore, users are able to adjust their preferred smoothness level in IClinfMRI.

In addition to spatial smoothing, inclusion/exclusion of global signal remains a controversial issue for rs-fMRI preprocessing. Previous studies of presurgical fMRI mapping have applied global signal as a nuisance regressor to reduce the spurious variance from non-neural sources and to improve the spatial specificity of the resulting functional networks, such as motor (Rosazza et al., 2014) and language network (Mitchell et al., 2013; Lee et al., 2016). However, this nuisance regression has been criticized for introducing the artificial anti-correlations in seed-based correlation analysis (Fox et al., 2009; Murphy et al., 2009). In the *Resting-state fMRI* module of IClinfMRI, regressing out global signal was built in as an optional preprocessing step upon user's decision.

Determining the threshold across individual mappings has been no consensus yet in the neuroimaging field because the various tasks result in different statistical sensitivity (Blatow et al., 2011; Nadkarni et al., 2015). Considering the fMRI mapping in clinical practice, the statistical threshold is determined by experienced clinicians who adjust a continuum of the threshold to obtain adequate activation extent without spurious clusters outside the eloquent cortex (Rosazza et al., 2014; Nadkarni et al., 2015). In contrast to such rater-dependent procedure, Lu et al. recently addressed an automatic procedure of threshold determination based on the training results (Lu et al., 2017). Nevertheless, no gold standard is reached for threshold determination at the current stage. Caution should be exercised when interpreting the statistical maps. In IClinfMRI, independent panels of threshold determination for both parametric and CVR maps were built for users to decide an appropriate threshold.

A limitation of IClinfMRI is that it calls functions in other free software including dcm2nii, AFNI, and SPM. Since AFNI is designed to run on Unix or Mac OS, those using a Windows operating system must install a virtual machine to run IClinfMRI.

In conclusion, by integrating fMRI techniques and implementing data conversion modules, our toolbox is a strong research tool that has been designed for translation to clinical practice. Two approaches in producing guidance maps—task-fMRI activation and the RH+MA map—were implemented for seed-based rs-fMRI mapping. By assisting in interpreting the clinical fMRI study, CVR mapping is able to provide visualization for indicating the potential false-negative areas in fMRI results. Any mapping result in the NIfTI format generated by either IClinfMRI or other research software can be exported in a DICOM format that is ready to be incorporated into PACS. IClinfMRI has been developed to incorporate advanced fMRI methods with streamlined processing and has shortened the processing time for presurgical mapping and other clinical applications. The software is freely available and can be requested by contacting the authors of this article.

## Author contributions

A-LH, PH, JJ, JH, and H-LL: contributed to the conception and design of the software; JJ, KN, SP, SF, VK, and DS: contributed to the preparation and interpretation of clinical data; A-LH, CW, J-HC, and H-LL: contributed to the preparation and revision of the manuscript. All authors reviewed and approved the manuscript.

### Conflict of interest statement

The authors declare that the research was conducted in the absence of any commercial or financial relationships that could be construed as a potential conflict of interest.
